# A new prokaryotic expression vector for the expression of antimicrobial peptide abaecin using SUMO fusion tag

**DOI:** 10.1186/s12896-019-0506-x

**Published:** 2019-02-15

**Authors:** Da Sol Kim, Seon Woong Kim, Jae Min Song, Soon Young Kim, Kwang-Chul Kwon

**Affiliations:** 1 Department of Biological Sciences, Andong National University, Andong, South Korea; 2 Department of Global Medical Science, Health & Wellness College, Sungshin University, Seoul, South Korea; 3 MicroSynbiotiX Ltd, 11011 N Torrey Pines Rd Ste. #135, La Jolla, CA 92037 USA

**Keywords:** Abaecin, Antimicrobial peptide, Small ubiquitin-related modifier (SUMO), Codon optimization, *Escherichia coli*

## Abstract

**Background:**

Despite the growing demand for antimicrobial peptides (AMPs) for clinical use as an alternative approach against antibiotic-resistant bacteria, the manufacture of AMPs relies on expensive, small-scale chemical methods. The small ubiquitin-related modifier (SUMO) tag is industrially practical for increasing the yield of recombinant proteins by increasing solubility and preventing degradation in expression systems.

**Results:**

A new vector system, pKSEC1, was designed to produce AMPs, which can work in prokaryotic systems such as *Escherichia coli* and plant chloroplasts. 6xHis was tagged to SUMO for purification of SUMO-fused AMPs. Abaecin, a 34-aa-long antimicrobial peptide from honeybees, was expressed in a fusion form to 6xHis-SUMO in a new vector system to evaluate the prokaryotic expression platform of the antimicrobial peptides. The fusion sequences were codon-optimized in three different combinations and expressed in *E. coli*. The combination of the native SUMO sequence with codon-optimized abaecin showed the highest expression level among the three combinations, and most of the expressed fusion proteins were detected in soluble fractions. Cleavage of the SUMO tag by sumoase produced a 29-aa-long abaecin derivative with a C-terminal deletion. However, this abaecin derivative still retained the binding sequence for its target protein, DnaK. Antibacterial activity of the 29-aa long abaecin was tested against *Bacillus subtilis* alone or in combination with cecropin B. The combined treatment of the abaecin derivative and cecropin B showed bacteriolytic activity 2 to 3 times greater than that of abaecin alone.

**Conclusions:**

Using a SUMO-tag with an appropriate codon-optimization strategy could be an approach for the production of antimicrobial peptides in *E.coli* without affecting the viability of the host cell.

**Electronic supplementary material:**

The online version of this article (10.1186/s12896-019-0506-x) contains supplementary material, which is available to authorized users.

## Background

The overuse of antibiotics for the last several decades and the prevalence of antibiotic-resistant bacterial infections present a threat to global health. About 30 million people are projected to be killed by antibiotic-resistance bacteria by 2050 [1]. Therefore, the discovery of novel antibiotic agents is of increasing medical importance. However, there is also a potential that over-use or abuse of a new antibiotic will give rise to more antibiotic-resistance bacteria. Growing difficulties from the discovery of new antibiotics to their clinical use have diverted attentions to a new therapeutic agent, antimicrobial peptides (AMPs) [2].

As an alternative approach to prevent the spread of bacteria which are resistant to antibiotics, antimicrobial peptides (AMPs) have been extensively studied since they have a broad spectrum of anti-infective activity against pathogenic bacteria with relatively low minimal inhibitory concentrations and with a property less capable of incurring resistance than conventional antibiotics, due to their nonspecific interaction with bacterial membranes, and their ability to work on multiple targets. In addition to the direct antimicrobial activities, many AMPs are associated with immunomodulatory properties as seen in host-defense peptides [3, 4].

To meet the demand of AMPs for clinical applications, the produciton of AMPs can be achieved by chemical synthesis. For example,  phase peptide synthesis approaches can provide easy isolation of peptides with high purity and less use of solvents by avoiding chromatographic purification [5]. However, chemical synthesis has several disadvantages that prevent cost-effective, industrial-scale production of AMPs. In addition, the synthesis of longer peptides with more than 50 amino acids is not favored [6].

For high production of large peptides, biological systems such as bacteria and yeast have been preferably used as production platforms [7–13]. Although these biological systems don’t need expensive active pharmaceutical ingredients (APIs) and toxic chemical solvents, there are still some issues remaining, such as the toxicity of the expressed AMPs to host cells, expensive purification, and low yield. As a solution to address these issues, plants can be a viable alternative expression platform [14–17]. In addition, they can be used as a delivery platform as well [18, 19]. Here, we report a new vector system for the production of AMPs, which can express the AMPs in both bacteria and plants.

The vector is designed to be operable in prokaryotic systems, and can be used to transform chloroplasts, a prokaryotic organelle of plants, for the large scale production of AMPs. The plant expression system can offer several other advantages over microbial expression systems, including no risk of endotoxin contamination, and oral delivery of bioencapsulated therapeutics using edible plants [18–21]. The expression cassettes of the vector were designed to be surrounded by flanking sequences, allowing the cassettes to be integrated into the chloroplast genome via double homologous recombination. Moreover, the copy number of the transgene in chloroplasts can be multiplied up to 10,000 per single plant cell [22, 23], leading to high expression of the transgene.

In addition, the vector was designed for AMPs to be expressed in a fused form using tags such as SUMO and 6xHis in order to eliminate the toxicity of AMPs to prokaryotic hosts, prevent protease degradation, increase solubility, and simplify purification and detection [24]. However, the tags need to be removed prior to clinical use. The final product needs to be an authentic or native amino acid sequence, so a cleavage recognition site should be placed between the therapeutic peptide and the tag. However, the removal of tag proteins at the cleavage site is often sterically hindered, and the cleavage action by proteases such as factor Xa, enterokinase, thrombin and tobacco etch virus protease, is often non-specific [24]. Unlike proteases that recognize linear amino acid sequences, the SUMO protease (sumoase) recognizes the tertiary structure of SUMO and prevents erroneous cleavage events in the target protein [24–26].

To evaluate our expression platform for AMPs in *E. coli*, abaecin from honeybee *Apis mellifera* was expressed using our new vector system. Abaecin is an important peptide in the bee innate immune system, and is found in a variety of bees including *Apis mellifera* [27], *Bombus pascuorum* [28] and *Bombus ignitus* [29]. As a proline-rich, non-glycosylated antimicrobial peptide [27], abaecin has a broad spectrum of bacteriolytic activity [30] and shows increased inhibitory effects on bacterial growth when treated with pore-forming peptides, such as cecropin A [13, 31], stomoxyn, and hymenoptaecin. The 34-aa-long cationic peptide contains 10 prolines (29%) with no cysteine residue, and the uniformed distribution of the proline residues through the entire peptide prevents the α-helical conformation [12, 27].

Here, we describe a new expression platform for the efficient produciton of AMPs, which can be potentially operable in both bacteria and plant chloroplast, and its use in an expression platform such as *E.coli* was first tested in this study. The new expression vector was built on the pUC19 backbone vector by assembling component DNA fragments, such as the promoter/5’ UTR, 3’ UTR and flanking sequences, which were derived from both plant chloroplast or bacterial genes, and further equipped with tagging systems such as SUMO and 6xHis. 

## Results

### Expression vector design and its construction

**Fig. 1:**
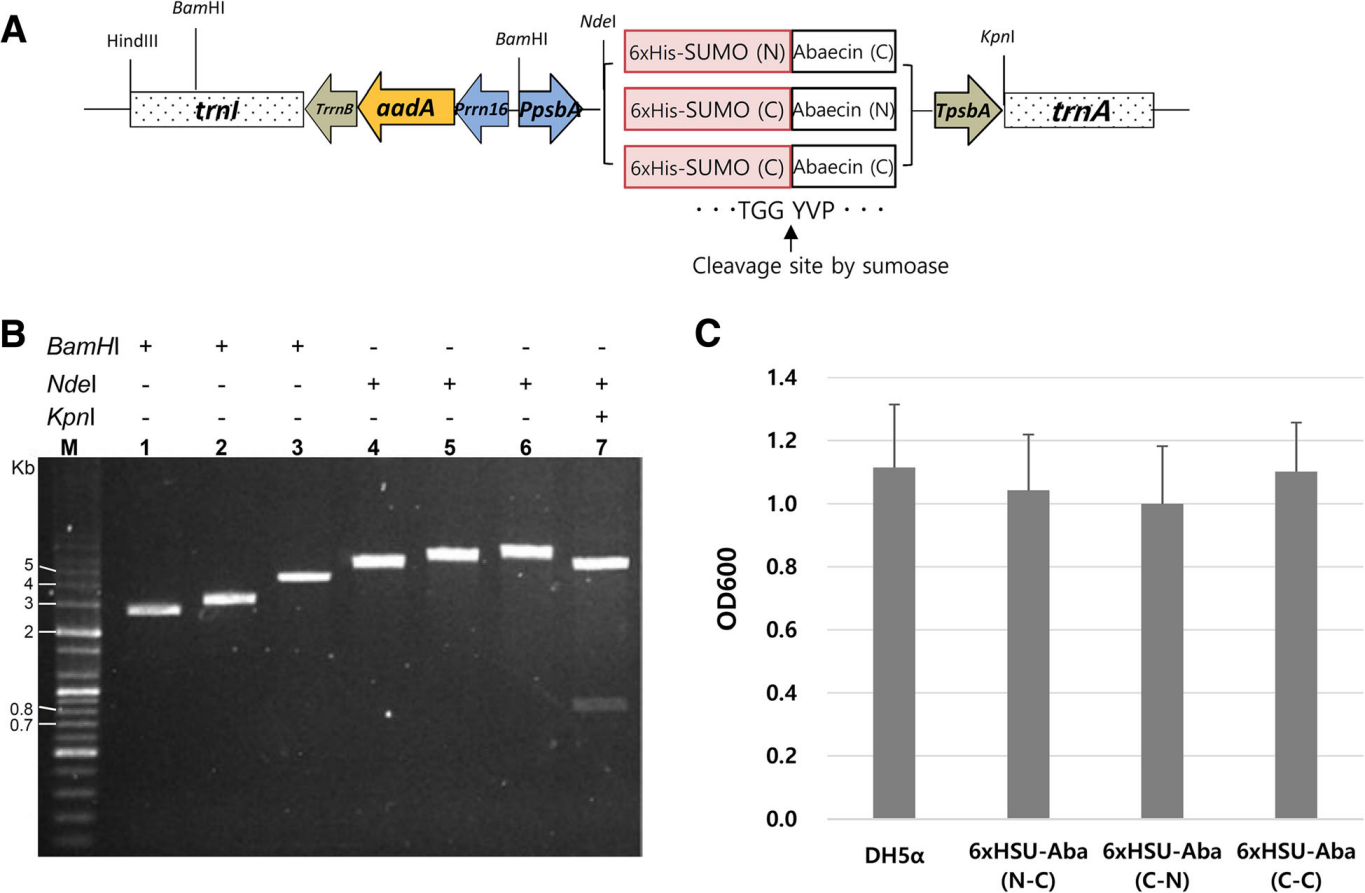
Construction of the expression vector and evaluation of the toxicity of 6xHisSUMO-abaecin fusion proteins to host cells. **a** Schematic diagram of the expression vector, pKSEC1. P*rrn* 16, 16S rRNA promoter; *aad*A, aminoglycoside 3′ adenylyltransferase gene; T*rrn*B, 3’ UTR of *E.coli* *rrn*B operon; P*psbA*, *psbA* promoter and 5’UTR; T*psb*A, 3’ UTR of the *psb*A gene; *trn*I, isoleucyl-tRNA; *trn*A, alanyl-tRNA. Three different fusion genes were cloned under the control of P*psb*A. N, native sequence; C, codon-optimized sequence. The cleavage junction between SUMO and abaecin, which is recognized by sumoase, is presented. **b** Restriction mapping for the confirmation of vector construction of 6xHisSUMO-abaecin fusion gene. M, DNA size marker; Lane 1, digestion of pUC19 backbone vector by *Bam*HI (2686 bp); Lane 2, insertion of P*psbA*-T*psbA* DNA fragment into the digested pUC19 and digestion by *Bam*HI (3105 bp); Lane 3, insertion of P*rrn*16-*aad*A-T*rrn*B DNA fragment into the intermediate vector of lane 2 and digestion of the plasmid (P*rrn*16-*aad*A-T*rrn*B-P*psb*A-T*psb*A:pUC19) by *Bam*HI (4576 bp); Lane 4, insertion of *trn*I DNA fragment into the plasmid of lane 3 and digestion of the plasmid (*trn*I-P*rrn*16-*aad*A-T*rrn*B-P*psb*A-T*psb*A:pUC19) by *Kpn*I (5343 bp); Lane 5, construction of a new expression vector, pKSEC1, by the insertion of *trn*A DNA fragment into the plasmid of lane 4 and digestion of the vector (*trn*I-P*rrn*16-*aad*A-T*rrn*B-P*psb*A-T*psb*A-*trn*A:pUC19) by *Kpn*I (6119 bp); Lane 6, cloning of 6xHisSUMO-abaecin into pKSEC1 and confirmation of the insertion by a single digestion with *Kpn*I (6527 bp) and by double digestion with *Kpn*I and *Nde*I (5708 bp and 819 bp) in lane 7. **c** Evaluation of toxicity of SUMO-abaecin fusion to host cells. Optical density (OD) values of overnight liquid cultures were compared between untransformed and transformed *E.coli* with three different 6xHisSUMO-abaecin:pKSEC1 constructs. Colonies of untransformed and transformed *E.coli* with each of the three different constructs were randomly picked from solid media and cultured in liquid media at 37 °C overnight and then OD values were measured at 600 nm next day. For the transformed *E.coli*, the liquid cultures were grown in both 100 μg/μl ampicillin and 50 μg/μl spectinomycin, while the untransformed one was cultured with no antibiotics. Each bar represents the mean and standard deviation values of three independent OD measurement experiments. DH5α, untransformed control; 6xHSU-Aba, 6xHisSUMO-abaecin; N, native sequence; C, codon-optimized sequence

A new expression vector was designed for use in prokaryotic systems such as bacteria and plant chloroplasts. The *psb*A promoter/5’ UTR and 3’ UTR were employed to drive the high expression of transgenes and stabilize the expressed transcripts, respectively [32, 33] (Fig. 1a). A second expression cassette, which can counteract spectinomycin, was constructed to select transformed cells. The antibiotic resistance gene, aminoglycoside-3″-adenylyltransferase (*aad*A), was put under the control of 16S rRNA promoter, and the transcripts were stabilized using the 3’ UTR fragment of the *E.coli* *rrn*B operon [34, 35]. For chloroplast expression of transgenes, flanking sequences which can integrate the expression cassettes into the chloroplast genome at a specific location were added to both ends of the combined expression cassette fragments. The flanking sequences were derived from the *trn*I and *trn*A regions of the tobacco chloroplast genome. The integration region is transcriptionally active and has been widely used for high expression of transgenes [36, 37]. All of the DNA elements which were amplified by PCR or synthesized were combined into the pUC19 backbone vector to construct a new vector, pKSEC1 (Fig. 1a, b).

The vector was further modified by the addition of tagging systems. SUMO, derived from human SUMO1, was attached to abaecin to increase solubility and prevent toxicity of abaecin to host cells. Also, a 6xHis purification tag was added to the N-terminus of SUMO to facilitate the purification of the SUMO-fused abaecin, and to isolate abaecin from SUMO after cleavage by sumoase. Three different combinations of codon-optimized synthetic sequences of the 6xHisSUMO-abaecin were cloned into the new vector, and their expression in an *E. coli* system was investigated after the confirmation of their sequences (Fig. 1a, b and Additional file 1: Figure S1). For the codon optimization, codon adjustment was performed according to a previous study [33], in which a new algorithm for codon optimization was developed based on the codon usage hierarchy of chloroplast *psb*A genes, and the codon-optimized sequences under the control of *psb*A/5’ UTR showed increased expression levels over their respective native sequences in both *E. coli* and plant chloroplasts. The expressions of all the three fusion constructs didn’t show any toxicity to host cells. As shown in Fig. 1c, there was no significant difference of optical density (OD) values between untransformed and transformed cells with the three fusion constructs, which were grown overnight at 37 °C.

### Evaluation of expression of His-tagged SUMO fused abaecin in *E. coli*

**Fig. 2:**
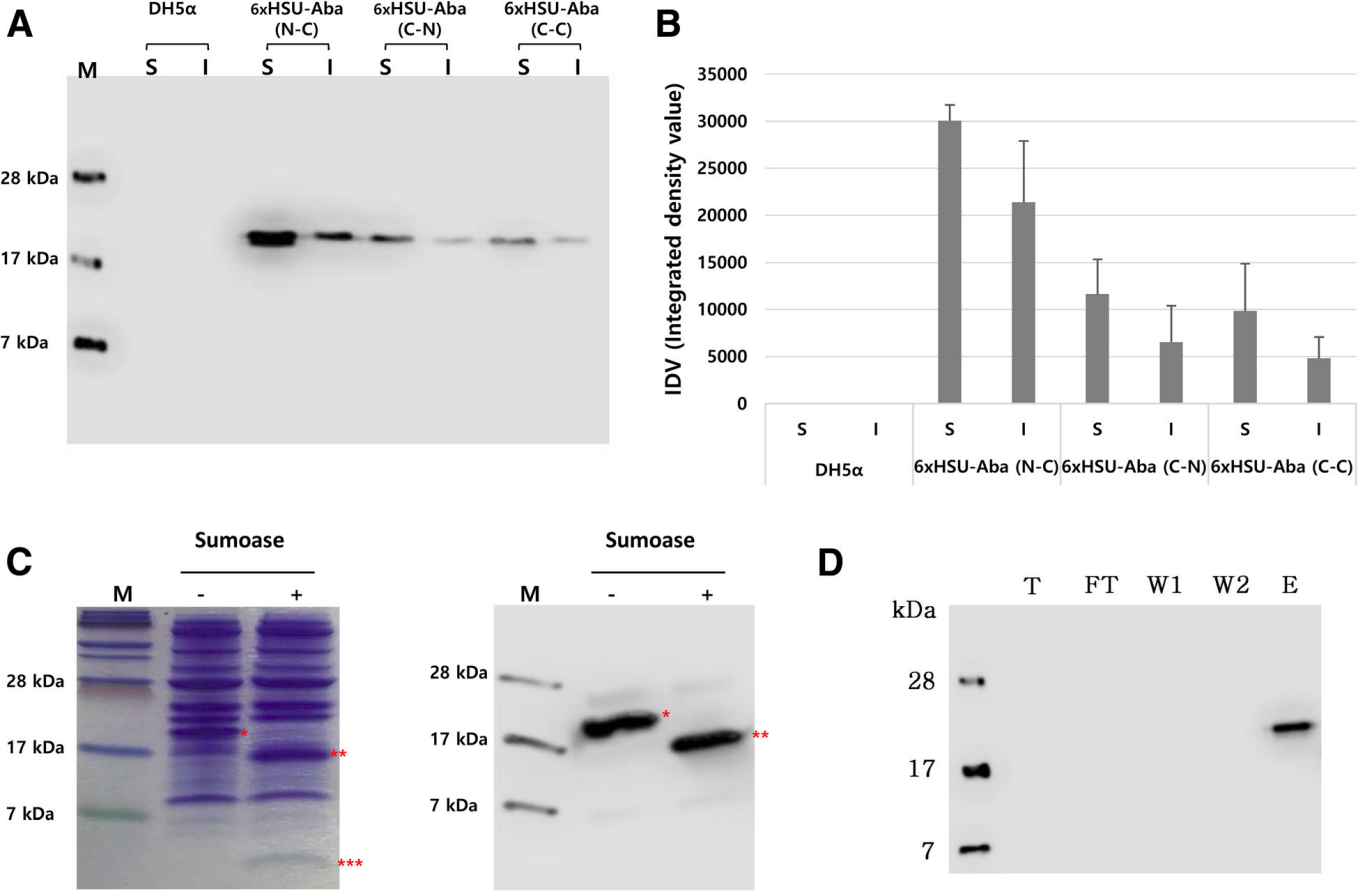
Western blot analysis for the comparison of expression levels between three different codon-optimized 6xHisSUMO-abaecin DNA constructs in *E.coli*, sumoase assay, and purification. ** a** Comparison of the level of expression between three different combinations of 6xHisSUMO-abaecin fusion protein using western blot. H_6_SU-Abe, 6xHisSUMO-abaecin; N, native sequence; C, codon-optimized sequence; S, soluble fraction; I, insoluble fraction; DH5α, untransformed *E. coli;* M**,** protein molecular size marker. The fusion proteins were detected using anti-His antibody. Each lane was loaded with 20 μg of protein. **b** Comparison of band intensities detected in (**a**). The band intensities from 5 independent western blot images were extrapolated using ImageJ software and represented with standard deviation. **c** Cleavage assay of the 6xHisSUMO (N)-abaecin (C) by sumoase. Coomassie staining assay (left panel) to detect cleavage products. Western blot assay (right panel) to investigate the cleavage efficacy of SUMO from 6xHisSUMO-abaecin by sumoase. *, 6xHisSUMO-abaecin; **, cleaved 6xHisSUMO; ***, cleaved abaecin; −, no treatment of sumoase; +, 6 h treatment of sumoase. Each lane was loaded with 20 μg of protein. **d** Western blot assay for the purified 6xHisSUMO-abaecin from *E. coli* using gravity Ni column. T, total protein; FT, flow-through; W, wash; E, elution

The three constructions of the 6xHisSUMO-abaecin fusion protein were evaluated using an *E. coli* expression system. Transformed *E. coli* cells with the three constructs were grown in liquid culture and the relative expression levels between the three fusion proteins were examined using an immunoblot assay with anti-His antibody. The expressed fusion proteins were detected around 20 kDa, not at 15.7 kDa which is a deduced molecular weight (Fig. 2a). This kind of discrepancy is very often observed in proline-rich proteins expressed in *E. coli*, due to presumably increased rigidity caused by high proline content, leading to slower migration than the same molecular weight protein [38–41]. Abaecin (3.9 kDa, 34 amino acids) contains 10 prolines in total of 34 amino acids, accounting for 34.9%, so it is assumed that the high content of proline residues affected the migration of all three fusion proteins (136 amino acids in length for each construct). Among three fusion proteins, 6xHisSUMO (N)-abaecin (C) (N and C, stand for “native sequence” and “codon-optimized sequence”, respectively) showed the highest expression, with 2.8 or 3.5 fold higher expression than 6xHisSUMO(C)-abaecin (N) or 6xHisSUMO(C)-abaecin(C), respectively (Fig. 2b). All three constructs showed that the expressed fusion proteins were detected at levels 1.4 to 2.1 times higher in the soluble fraction than in the insoluble one (Fig. 2b).

From these data, we confirmed that the newly designed vector is operable in a prokaryotic system and that the native sequence of the heterologous human SUMO gene improved the expression of the SUMO-fused abaecin in *E. coli* (Fig. 2a).

### Cleavage of abaecin from purified His-tagged SUMO-abaecin and MALDI-TOF analysis

The cleavage efficiency of sumoase on the fusion protein was investigated by treating the soluble proteins extracted from *E. coli* with sumoase for 6 h and then detecting the treated proteins with Coomassie staining and western blot. As seen in Fig. 2c, the fusion proteins detected at 19 kDa before treatment of sumoase were cleaved and produced two products (Fig. 2c). To confirm the result, an immunoblot assay with a replicate gel was performed using anti-His antibody. The band for the cleaved-off 6xHisSUMO domain was detected around 16 kDa, along with a band for the fusion protein around 19 kDa (Fig. 2c). This data confirmed that the tertiary structure of SUMO was correctly formed in *E. coli* and recognized by sumoase (Fig. 2c).

**Fig. 3:**
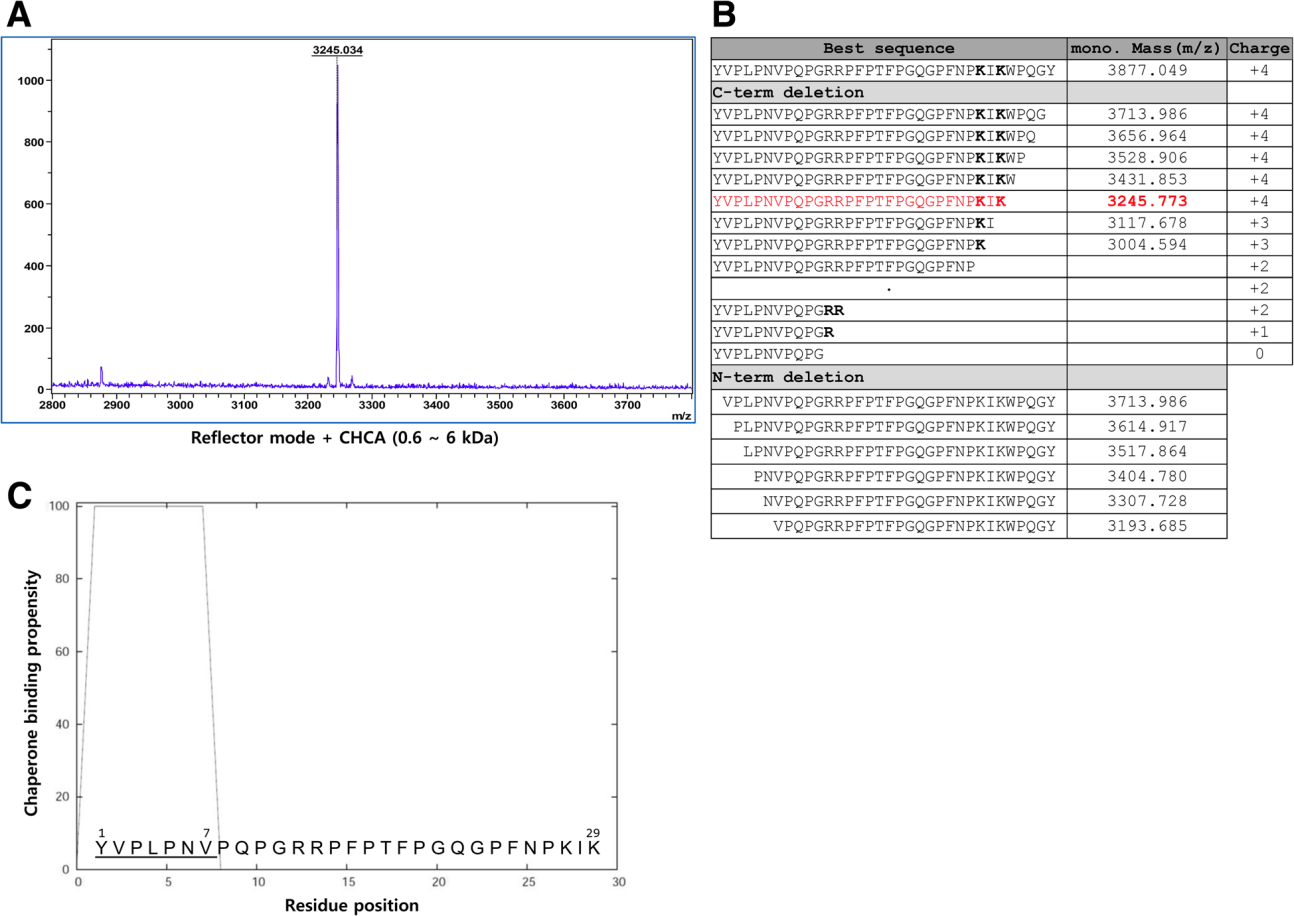
MS analysis of the cleaved-off abaecin using MALDI-TOF and investigation of binding sequence of 29-aa-long abaecin to DanK. **a** MS spectra obtained from reflector mode. The reflector mode was performed using α-cyano-4-hydroxycinnamic acid (CHCA) matrix for the lower range of mass from 0.6 to 6 kDa to analyze the target peak at high resolution. **b** Table of masses and overall charge for a series of deleted peptide sequences from abaecin. Masses were analyzed for each deleted peptide sequence from either N- or C-termini in order to find the best peptide sequence matchable to the detected mass (m/z), 3245.034. Net charges at pH 7 were calculated by Peptide Property Calculator (https://www.biosyn.com/peptidepropertycalculatorlanding.aspx).The amino acids which affect the overall charge of abaecin were represented as bold fonts, such as K for Lys and R for Arg.  **c** Sequence analysis for the binding affinity of the 29-aa-long abaecin to DnaK. The amino acid sequence of the 29-aa-long abaecin is represented, and the putative binding sequence to DnaK is underlined and numbered

With confirmation of the proper expression of 6xHisSUMO-abaecin and cleavage of SUMO tag (Fig. 2c), the fusion proteins were purified for mass analysis of abaecin cleaved from the fusion protein (Fig. 2d). The abaecin cleaved by sumoase was examined by MALDI-TOF analysis to verify the precise cleavage between SUMO and abaecin. Singly protonated molecular ion (M + H) + of the cleaved-off abaecin was observed at m/z 3245.034 Da using reflector mode (Fig. 3a), while the theoretical molecular mass is 3877.049 Da (Fig. 3b). This discrepancy between the observed and theoretical values suggests the possibility of deletion of abaecin peptide at either N- or C- termini. To identify the peptide peak, we calculated the masses for a series of deleted abaecin peptides from either N- or C-termini and compared the theoretical masses to the observed mass (Fig. 3b) The best amino acid sequence corresponding to the observed mass at 3245.034 Da was deduced as YVPLPNVPQPGRRPFPTFPGQGPFNPKIK, which indicates the deletion of 5 amino acids from C-terminus of abaecin (Fig. 3b). Based on the results, although the proper cleavage between SUMO and abaecin occurred, undesired cleavage happened at the C-terminus.

Since the small cationic peptides are very susceptible to proteolytic degradation in *E.coli* [10], the positive charge distribution of the abaecin was analyzed (Fig. 3b) from which the positive charge patch created by two Lys (K) residues at C-terminus was likely targeted by proteolytic attack.

Next, we analyzed whether or not the 29-aa-long abaecin still retained the functional binding sequence to its intracellular target molecule, DnaK. For the analysis, we used limbo server (http://limbo.switchlab.org) [42] and found that the shorter form of abaecin still had a binding sequence to the DnaK; YVPLPNV, numbered from 1 to 7 (score 1.65) (Fig. 3c). Therefore, we continued to test an antimicrobial activity of the 29-aa-long abaecin derivative with or without cecropin B against *Bacillus subtilis*.

### Antibacterial activity of purified abaecin against *Bacillus subtilis*

**Fig. 4:**
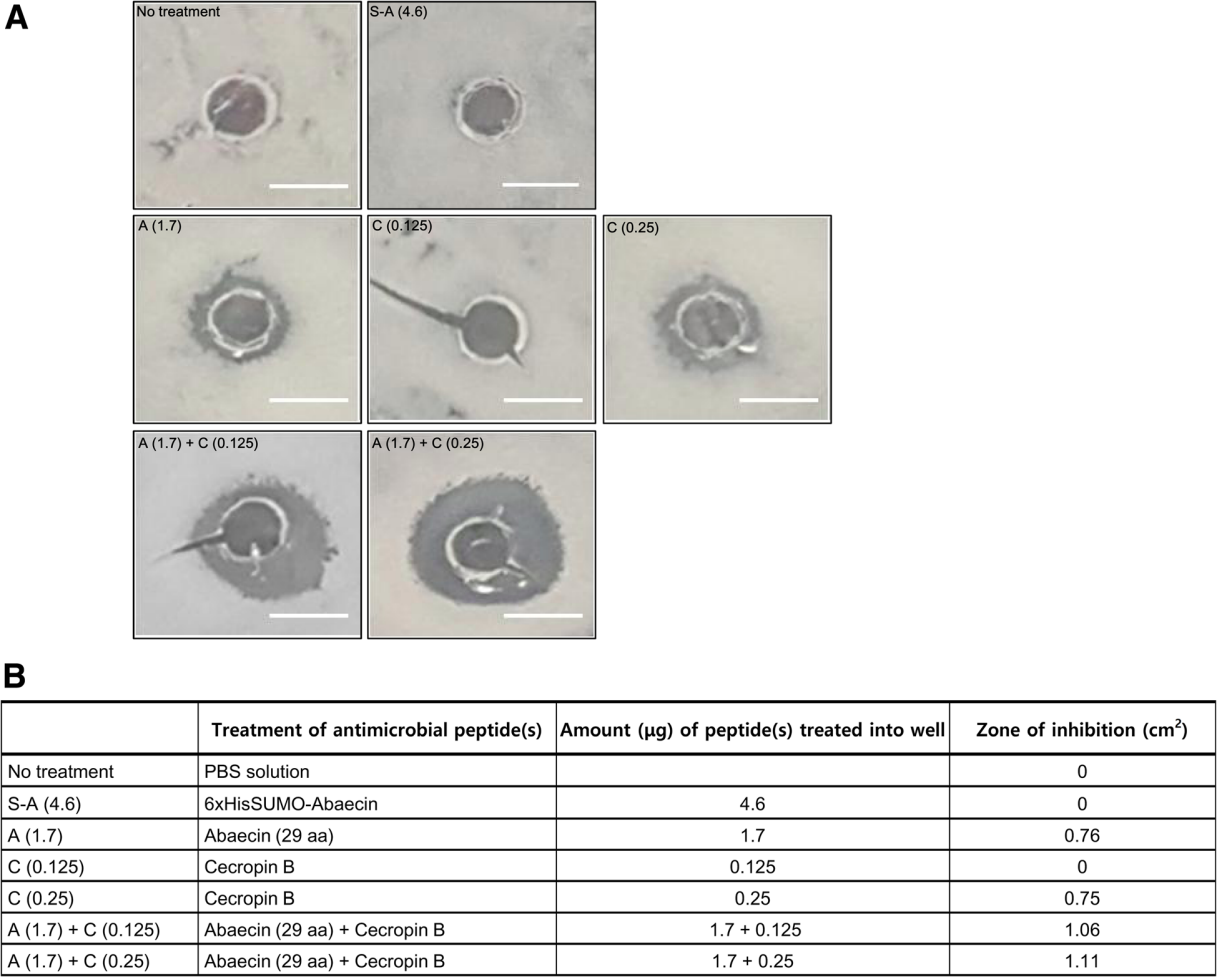
Evaluation of antibacterial activity of 29-aa-abaecin with or without cecropin B. **a** The antibacterial activities of 29-aa abaecin with or without cecropin B against *B. subtilis.* Agar plates spread with 100 μl of *B. subtilis* liquid culture grown overnight were punctured and dropped with 10 μl of abaecin or cecropin B or both peptides, which were purified and resuspended in 1X PBS, then incubated at 37 °C for 16 h. The antimicrobial peptides and their amount treated into the corresponding wells are represented in (**b**). **b** Evaluation of inhibitory effect by antimicrobial peptides on *B. subtilis* by measuring the inhibited zone area. The inhibited zone areas of *B. subtilis* were extrapolated using ImageJ software. Scale bars represent 1 cm

To evaluate the antimicrobial activity of 29-aa abaecin expressed in *E. coli*, the activity was tested against *B. subtilis* using an agar diffusion assay with the purified abaecin derivative. We also tested whether the SUMO-fused abaecin is toxic to bacteria when treated externally, although we had already confirmed that there was no toxicity of the 6xHisSUMO-fused abaecin to host cells when expressed in the cells (Fig. 1c). The treatment of the 6xHisSUMO-abaecin didn’t show bacteriolytic activity against *B. subtilis* (Fig. 4a, b). In the preliminary test, we found that anti-*B. subtilis* activity of 29-aa abaecin required an amount of 1.7 μg (Fig. 4a, b). The antimicrobial activity of 29-aa abaecin was enhanced in a combination treatment with cecropin B. No antimicrobial activity was observed by cecropin B when 0.125 μg of the peptide was treated (Fig. 4a, b), however, the antimicrobial activity of abaecin, at the dose of 1.7 μg, was increased by 40% when combined with the cecropin B (0.125 μg) (Fig. 4a, b). The antibacterial activity of abaecin was further enhanced by the increase of cecropin B quantity up to 0.25 μg (Fig. 4a, b). These results are consistent with the previous reports that the functional interaction of abaecin with other pore-forming peptides, such as cecropin A, stomoxyn and hymenoptaecin, at their sublethal concentrations, reciprocally potentiates bacteriolytic activity by increasing membrane permeabilization [13, 31, 43].

Taken all together, 6xHisSUMO-tagged abaecin expressed in a soluble form in *E. coli* caused no toxicity to the host cells, and the tag was properly recognized and removed by sumoase, but the cleaved abaecin was 29-aa long in size, not 34-aa long. However, the purified 29-aa-long abaecin retained its antimicrobial activity and the activity was potentiated by co-treatment with a pore-forming antimicrobial peptide, cecropin B.

## Discussion

The limits of conventional antibiotics in treating pathogenic microbes and the increasing prevalence of antibiotic-resistant pathogens have led to the exploration of viable alternatives, including antimicrobial peptides. Only a limited number of antibiotics are available for clinical use, and they have similar activity spectrum and action mode [44, 45]. AMPs have distinct advantages over antibiotics such as remarkable structural and functional diversity, and immunomodulatory activity. Some AMPs show a broad range of action, which can be effectively used to treat multi-microbial infections including both Gram-positive and Gram-negative bacteria [45, 46].

Currently, no antibiotic peptides are available for clinical use. However, a number of AMPs are under clinical trials and development, and their applications are not limited to directly killing pathogens: e.g. pexiganan (for the treatment of bacterial infections, to diabetic foot ulcers), omiganan (catheter infections and rosacea), hLF-11 (bacterial and fungal infections in immunocompromised stem cell transplantation), novexatin (fungal infections), CZEN-002 (vaginal candidiasis), LL-37 (wound healing), PXL01 (prevention of post-surgical adhesion formulation), Iseganan (oral mucositis), and PAC-113 (oral candidiasis) [45].

Despite their potential, the development and availability of AMPs for clinical use is met with several challenges. Primarily, the high production cost and low scalability of chemical peptide synthesis prevent the widespread development and adoption of AMPs as a viable clinical treatment [6]. Biological production using recombinant prokaryotic systems is a viable alternative to chemical synthesis, but issues such as toxicity to host cells, degradation of the product by protease, and low yield must be addressed [47].

In this study, a SUMO tagging system was used to prevent toxicity of the expressed antimicrobial peptide to host cells. The commonly used fusion carriers such as thioredoxin (12 kDa) and glutathione-S-transferase (GST, 26 kDa) have several advantages associated with increased solubility, promotion of proper folding and prevention of toxicity of AMPs [9, 46, 47]. However, GST increases the relative molecular weight ratio of carrier proteins to the peptides, which leads to low AMP yields. Also, several GST fused-AMPs expressed in *E.coli* showed proteolytic activity, resulting in inefficient or failed AMP productions. Thioredoxin is more favorably used for peptide production than the GST due to its small size, allowing high peptide yield attributed to the high peptide-to-carrier ratio [47]. However, the proteases used for the release of attached peptides from the carriers are more expensive and more sensitive to pH and chaotropes when compared to SUMO protease [47]. Moreover, AMPs tagged to aggregation-promoting carriers such as PurF fragment, PaP3:30 and ketosteroid isomerase have shown toxicity to host bacterial cells [9, 48].

The SUMO tag has many similar advantages to the thioredoxin or/and GST systems including high yield resulting from the high ratio of peptide-to-tagging protein, enhanced solubility, and no toxicity to host cells, but it also has unique advantages over other tags. Sumoase can recognize the tertiary structure of SUMO and cleave it from substrates with no attachment of unwanted amino acids to peptides [24–26]. The precise cleavage of target proteins from SUMO fusions by sumoase is confirmed by comparing the molecular mass and N-terminal amino acid sequence of the released peptides to the corresponding synthetic peptides using matrix-assisted laser desorption/ionization time-of-flight mass spectrometry (MALDI-TOF) and Edman degradation. More detailed information about the current tagging and expression systems for the production of antimicrobial peptides in *E.coli* is included in Additional file 2: Table S1.

In this study, we confirmed the proper cleavage of abaecin from SUMO using MALDI-TOF, but there was 5-aa-long C-terminal deletion from the abaecin. In contrast to our expectation, the attached SUMO tag (overall charge − 5) didn’t protect the abaecin (overall charge + 4, Fig. 3b) via electrostatic interaction from endogenous proteases in an *E.coli* system. Small size peptides with high cationic content are highly susceptible to proteolytic attack in *E.coli* as shown in Piers et al.’s study [10]. In the study, direct expression of small size cationic peptide such as human neutrophil peptide 1 (HNP-1, a kind of defensin) wasn’t successful, while its transcript was detected. Even when the HNP-1 and other cationic peptide, synthetic cecropin/melittin hybrid (CEME), were fused to GST, the fusion proteins were proteolyzed. However, the proteolysis wasn’t observed when anionic pre-pro defensin sequence was inserted between the GST and the cationic peptides. Based on our analysis, a positively charged patch created by two Arg (R) at 12th and 13th positions (Fig. 3b) seemed to not be recognized by endogenous proteases possibly due to charge neutralization effect by the electrostatic interaction with SUMO, while the second patch created by two Lys (K) at 28th and 30th positions (Fig. 3b) seemed subjected to proteolytic attack. Another possibility of the C-terminal deletion could be a consequence of excessive sonication, resulting in denaturation of the fusion protein and breakdown at the C-terminus. Also, it can’t be ruled out that the sumoase could aberrantly cleave the abaecin. It is thought that sumoase is highly specific and active in a broad range of conditions; it cleaves effectively in a wide range of pH (5.5–10.5) and temperatures (4–37 °C), and even in detergents such as 2 M urea or 20 mM DTT or β-mercaptoethanol [25]. However, the cleavage study at the junction between SUMO and a partner protein showed that the cleavage didn’t work when the first amino acid of the partner protein was Pro (P) [25]. Also, the introduction of a stretch of several Try (W) residues close to the cleavage site caused random cleavage within the fusion protein due to an inaccessibility of sumoase by steric hindrance, which, however, was relieved by addition of 1 M urea and led to the release of correct size peptide [26]. Considering the high content of proline in abaecin, the repeated kinks generated by 10 prolines in the abaecin could create a steric hindrance when attached to SUMO, which would lead an unexpected random cleavage. To investigate these issues mentioned above, SUMO could be further modified in a way which introduces additional glycine resides at the cleavage junction to release steric hindrance. In our unpublished study, cleavage of SUMO-cecropin B at the junction by sumoase happened only after introduction of additional glycine residues. Also, genetically modified SUMO which has more negative charge could provide a better protection to cationic peptides.

Although the C-terminus of abaecin sustained a 5-aa deletion after sumoase cleavage, the 29-aa-long abaecin showed antimicrobial activity against *B. subtilis* in the functionality study, while the SUMO-abaecin fusion protein had no bacteriolytic activity as shown in Fig. 4. Furthermore, the bacteriolytic activities were consistent with previous reports in which the antimicrobial activity of abaecin alone was further enhanced with combinatory treatment with other pore-forming AMPs [31, 43]. Although abaecin has high binding efficacy to DnaK like other proline-rich DnaK-binding AMPs including metalnikowins, metchnikowins, onocin Onc72, apidaecin Api88, drosocin and pyrrhocoricin, the abaecin hasn’t a conserved motif, YL/IPRP [43]. So we analyzed both abaecin amino acid sequences such as 34-aa and 29-aa-long abaecins for the functional binding sequence to DnaK using limbo server and found that the full length abaecin has two binding sequence sites: one is at N-terminus (^1^YVPLPNV^7^, score 1.7) and the other one is at C-terminus (^25^NPKIKWP^31^, score 2.8). So we assume that the reason why the 29-aa long abaecin still has antimicrobial activity (Fig. 4) is due to the presence of N-terminal binding sequence to DnaK (Fig. 3c).

The main purpose of this study was to introduce a new expression platform for the production of antimicrobial peptides in *E.coli* system in a way that does not harm the host cell. Abaecin was chosen as a reference AMP because it targets the intracellular molecule DnaK, prokaryotic heat shock protein 70 (Hsp70), and the binding of abaecin to DnaK can cause protein metabolism to be compromised in *E. coli,* resulting in host cell death [43]. The 6xHisSUMO-abaecin fusion protein, however, did not exhibit toxicity to *E. coli* host cells. The transformed *E. coli* cells grew in liquid culture at the similar growth rate to untransformed ones (Fig. 1c). Furthermore, the purified fusion protein showed no anti-bactericidal activity against *B. subtilis* (Fig. 4a, b). The normal growth of the transformed *E. coli* and the lack of antimicrobial activity of the fusion protein to *B. subtilis* proved that the lethal activity of the AMP to the host cells can be properly shielded by SUMO but its activity can be restored by the release from the tag. The antimicrobial activity of AMPs is generally determined by the hydrophobicity and the net positive charge. As reported in the previous study, the electrostatic interactions between the positive charges (between + 4 and + 6) of AMPs and negatively charge residues within SUMO (overall charge − 5) seem to play a key role in neutralizing the bacteriolytic activity and protecting AMPs from degradation by endogenous proteases [26]. In this regard, although the protection of abaecin from proteolytic degradation by SUMO failed, the C-terminal deleted abaecin still had antimicrobial activity. Furthermore, the toxicity of abaecin was, in some way, successfully prevented by the SUMO tag via the presumed electrostatic interaction.

The SUMO tagging system is also advantageous because it has no disulfide bond. Due to the reducing environment of the *E. coli* cytoplasm, proteins that require disulfide bonds fail to achieve their active forms and ultimately form inclusion bodies or are degraded by proteases. To achieve and maintain their active forms, such proteins need to be redirected to periplasmic space or secreted, which are energy-consuming and could potentially lower target protein yield. However, the SUMO tagging system does not require the additional steps because it only contains one cysteine in its entire 96 amino acid sequence.

Overall, the SUMO-abaecin fusion proteins were detected more in soluble fractions than insoluble ones (Fig. 2a, b) with no toxicity to the host cells. There have been many reports that SUMO fusions increased the solubility of difficult-to-express proteins in *E.coli*, such as GFP, metalloprotease (MMP13) [25] and severe acute respiratory syndrome coronavirus (SARS CoV) proteins including 3CL protease, nucleocapsid protein and spike C protein [49, 50]. However, in contrast to antimicrobial peptides, those proteins are not toxic to the host cells. To conclusively distinguish the effect of SUMO on the solubility of the fusion protein, the expression of abaecin alone would need to be compared with SUMO-abaecin fusion protein, which, however, was not examined in this study due to technical issues such as possible low stability and potential toxicity to host cells. As mentioned above, the direct expression of the small size and high cationic content of AMPs are highly susceptible to endogenous proteolysis [10]. Furthermore, the expression of abaecin alone could be lethal to host cells because the peptide inhibits intracellular DnaK [43], which is a central organizer of the chaperone network in *E.coli*. The chaperone protein interacts with ~ 700 cytosolic proteins. Among them, ~ 180 proteins are relatively prone to be aggregation and rely extensively on DnaK during and after their initial folding [51].

The translation efficiency of codon-optimized heterologous sequences was evaluated in this study through partial or whole optimization in order to find the most accommodating nucleotide sequence in *E.coli* system. Codon usage was adjusted according to the codon usage preference of a gene, *psb*A, which is highly expressed in prokaryotic systems, and then three different combinations of codon-optimzed sequences were created. The codon adjustment was performed in a way to increase the compatibility between 5’ UTR of the *psb*A promoter and the 5′ coding region of the fusion gene, with an increase of AT content of the SUMO sequence to 63.5% from 59.5%. In contrast to our expectation, the codon optimization of N-terminal SUMO of the fusion protein failed to improve the expression level over the non-optimized counterpart. As seen in the Fig. 2a, b, the native sequence of SUMO performed better than its corresponding codon-optimized sequence for the expression of the fusion proteins. The expression level of 6xHisSUMO-abaecin (native – codon-optimized) was 2.8 or 3.5 times higher than that of 6xHisSUMO-abaecin (codon-optimized - native) or 6xHisSUMO-abaecin (codon-optimized – codon-optimized), respectively (Fig. 2b). This is likely due to the stability of the transcribed mRNA of the native SUMO sequence, which may be relatively higher than the stability of other codon-optimized sequences [52]. Another possible explanation is that the compatibility of the 5’ UTR of the promoter with the 5′ coding sequence of the codon-optimized SUMO could be compromised, resulting in an unstable or inefficient translational initiation complex [53, 54]. It is generally thought that translational efficiency is influenced by the efficiency of the formation of translational initiation complex and thus the first ~ 30–50 codons are considered more important than the rest of the sequence [55, 56]. Therefore, the marginal difference of expression level between codon-optimized SUMO fused native and codon-optimized abaecins could be a conseqeunce of the inefficient formation of the translational initiation complex.

Recent studies have found that the reciprocal functional interaction of abaecin with pore-forming peptides occurred not only with the peptides co-expressed in the same species, but with ones from other species [13, 31, 43]. Pores created in the membrane by the pore-forming peptide allow abaecin to access its intracellular target, DnaK. The inhibited heat shock proteins compromise protein metabolism, and the damaged pores remain unrepaired, thus allowing abaecin even greater access to its target. Abaecin’s ability to increase membrane permeabilization consequently reduces the minimal inhibitory concentrations of both abaecin and other pore-forming peptides in a reciprocal manner [31, 43]. Likewise, the activity of the pore-forming peptide cecropin B from *Hyalophora cecropia* was potentiated in a combinatorial treatment with abaecin (Fig. 4), showing that abaecin can be used with diverse pore-forming peptides to inhibit bacteria which are renitent to conventional antibiotics.

One of the challenges that the application of AMPs presents is the demand for effective and patient-friendly delivery system, particularly, for the patients with chronic diseases. Currently, most AMPs under clinical development are designed to target local infections using a topical formulation. Only a few AMPs are being developed for systemic delivery [45]. Although an oral delivery system is most likely preferred due to straightforward administration, AMPs require special consideration because peptides are rapidly broken down in the gastrointestinal tracts due to the high concentration of proteases and high acidity. In this respect, edible plants can be used as a delivery platform, by which peptide based drugs bioencapsulated within the plant cells can be protected from the harsh environment of the gastrointestinal tract. But the drugs can be released into the intestine by the break-down of the cell walls by cellulolytic bacteria. The plant expression and delivery system can also eliminate the concern of endotoxin contamination, which causes fatal septic shock to recipients. Furthermore, the oral delivery of peptides by edible plant cells eliminates the need for expensive downstream purifications, reducing product cost and benefiting patients [18, 20, 21]. In our future study, we will evaluate the efficacy of the plant expression system as well.

## Conclusions

Our SUMO tagging expression system showed an applicable method for the production of AMPs in *E. coli*, which although couldn't completely protect abaecin tested in this study from endogenous proteolytic degradation, did increase solubility and prevented toxicity of abaecin to host cells. Furthermore, the released AMP from the SUMO tag retained its antimicrobial activity and showed enhanced antimicrobial effects by combinatorial application with pore-forming peptides. Therefore, further studies for the enhancement of stability and translational efficiency of the SUMO-fused AMP will make the use of AMPs more affordable for clinics and patients.

## Methods

### DNA fragment amplification and synthesis

The recombinant protein/peptide coding sequences and DNA elements were designed referencing the deposited sequences in the National Center for Biotechnology Information (NCBI) or previous reports. The GenBank accession numbers for the sequences are as follows: Abaecin, NM_001011617.1; SUMO, NM_003352.4; Cecropin B, M34924.1. Codon-optimized nucleotide sequences such as abaecin and SUMO, and the native sequences of P*rrn*16, *aad*A and T*rrnB* deduced from a previous report ([57], AF327719.1) were synthesized by Macrogen (South Korea). The sequences of P*psb*A/5’ UTR ([58], EU520589.1) and T*psbA* ([59], AY442171.1) were deduced from previous reports and amplified using tobacco chloroplast genomic DNA (Z00044.2) as a template by PCR. Codon optimizations were performed according to a previous study [33].

The primers used for PCR amplification of P*psbA*, T*psbA*, *trn*A and *trn*I DNA fragments are as follows: P*psb*A-F, 5’-CCCGGGCAACCCACTAGCATATC-3′; P*psb*A-R, 5’-CCTCCTATAGACTAGGCCAGGATCTAGATTACATATGAAAATCTTGGTTTATTTAATCATCAGGG-3′; T*psbA*-F, 5’-CCCTGATGATTAAATAAACCAAGATTTTCATATGTAATCTAGATCCTGGCCTAGTCTATAGGAGG-3′ and T*psbA*-R, TCGAATATAGCTCTTCTTTCTTATTTCAATGATATTATT-3′. *trn*A-F, 5′- GGGGAAGAATTCGGGGATATAGCTCAGTTGGTAG-3′; *trn*A-R, 5′- GAAAAAGGTACCTGGAGATAAGCGGACTCGAACC-3′; *trn*I-F, 5′- GGGGAAAAGCTTGGGCTATTAGCTCAGTGGTAG-3′ and *trn*I-R, 5′- GAAAAAGTCGACTGGGCCATCCTGGACTTGAAC-3′.

### Construction of expression vector

P*psb*A-T*psb*A DNA fragments amplified by overlapping PCR were treated by *Ase*I and *Sal*I and inserted into pUC19 backbone plasmid restricted with *Nde*I and *Sal*I. The ligated plasmids were further digested with *Bam*HI and *Eco*RI. The DNA fragments of P*rrn*16-*aad*A-T*rrn*B were synthesized by Macrogen and digested with *Bam*HI and *Eco*RI, and were then ligated with the intermediate vector. The pUC19 vectors containing P*rrn*16-*aad*A-T*rrn*B-P*psb*A-T*psb*A were combined with *trn*I DNA fragments using *Hind*III and *Sal*I, and then *trn*A fragments were inserted using *Kpn*I and *Eco*RI. The newly constructed vector, pKSEC1, was used for the expression of recombinant fusion gene, 6xHisSUMO-abaecin, which was inserted into the vector by *Xba*I and *Nde*I.

### Expression of 6xHisSUMO-abaecin in *E. coli*

The *E. coli* BL21 transformed with 6xHisSUMO-abaecin:pKSEC1 were grown in 4 L Terrific Broth (Sigma-Aldrich, USA) containing 100 μg/mL of ampicillin and 50 μg/mL of spectinomycin at 37 °C for 3 h at a speed of 200 rpm and the overnight culture was further grown at 18 °C overnight. The cells were collected by centrifugation (8000 rpm, 4 °C, 3 min) and resuspended in 140 mL of buffer including 20 mM Tris, 300 mM sodium chloride and 5 mM Imidazol. Then the cells were sonicated by a cycle of 5 s on and 15 s off (SONICS VC505, USA) for 40 m, and soluble and insoluble fractions were separated using centrifugation (13,000 rpm, 4 °C, 1 h) then quantified using Bradford (Sigma-Aldrich, USA). The quantified protein samples were mixed with 2X Laemmli sample buffer (Bio-Rad, USA) and heated at 95 °C for 5 min. The heated proteins were run on SDS-PAGE and the separated proteins were blotted onto PVDF membrane. The membrane was blocked with 1X TBS-T (0.1% Tween 20 and 5% skim milk) for 1 h at room temperature. After that, anti-His antibody (Santa Cruz, USA), diluted 1:1000 in the blocking solution, was incubated at 4 °C for 16 h. The membrane was washed with 1X TBS-T buffer for 5 min thrice. Secondary antibody (goat anti-rabbit IgG-HRP, Santa Cruz, USA), diluted 1: 5000 in the blocking solution, was incubated at room temperature for 1 h. The washed membrane was subject to ECL buffer for development using C-DiGit Blot Scanner (Li-Cor, USA).

### Purification of the recombinant abaecin

The *E. coli* BL21 transformed with 6xHisSUMO-abaecin:pKSEC1 were grown and treated as described above. After sonication, the supernatant was filtered through filter paper (Advantec, Japan) and 0.45 μm syringe filter (Minisart syringe filter, Sartorius stedim biotech, Germany). The filtrate was incubated with His 60 Ni Suferflow resin (Takara, Japan) by inverting slowly for 1 h at 4 °C. After the binding incubation, the column was washed with 50 column volumes of wash buffer (20 mM Tris, 300 mM sodium chloride, 50 mM Imidazol, pH 8.0). 6xHisSUMO-abaecin fusion proteins bound to Ni resins were eluted approximately with 5 column volumes of elution buffer (20 mM Tris, 300 mM sodium chloride, 250 mM Imidazol, pH 8.0). Recombinant abaecin was isolated from 6xHisSUMO by treatment of SUMO protease (Enzynomics, South Korea) at 30 °C for 6 h. To confirm the cleavage, SDS-PAGE was performed using NuPAGE™ 4–12% Bis-Tris Gel (Invitrogen, USA) with MES buffer.

### Mass spectrometry

For MS analysis, the eluted samples were applied onto Anchor-Chip 600 targets (Bruker Daltonik, Germany) using α-cyano-4-hydroxycinnamic acid (CHCA) (Sigma, C2020) as matrix according to the manufacturer’s recommendations. MS measurement was performed on an autoflex II TOF/TOF. Mass spectrum was acquired in the reflector mode using CHCA matrix. The mass analysis was carried out by Life Science Laboratory. Co (http://www.emass.co.kr, South Korea).

### Antimicrobial activity

Agar diffusion assay was performed to evaluate the antimicrobial activity of abaecin. One hundred microliter of *B. subtilis* liquid cultured overnight was plated on LB agar medium and punctured using a tip then purified abaecin was dropped with or without cecropin B that was expressed and purified in the same way as described above in our lab. The plates were grown at 37 °C for 16 h and the zone areas, where the growth of *B. subtilis* was inhibited by abaecin, were measured using ImageJ software.

## Additional files

Additional file 1: **Figure S1.** Sequences of three different codon-optimized 6xHisSUMO-abaecin. (PDF 163 kb)

Additional file 2: **Table S1.** Expression of antimicrobial peptides using *E.coli* and their subsequent release and purification. (PDF 73 kb)

## Abbreviations

AMP: Antimicrobial peptide
Hsp70: Heat shock protein 70
SUMO: Small ubiquitin-related modifier 1
