# Supplementary material for: A new prokaryotic expression vector for the expression of antimicrobial peptide abaecin using SUMO fusion tag
Source: BMC Biotechnol. 2019 Feb 15;19:13. doi: 10.1186/s12896-019-0506-x (PMC6377777; doi:10.1186/s12896-019-0506-x)
Supplement: Supplementary file 1 — Figure S1. Sequences of three different codon-optimized 6xHisSUMO-abaecin. (PDF 163 kb) [file 12896_2019_506_MOESM1_ESM.pdf]

Supplementary Fig. 1

A Sequence for 6xHis(N) SUMO(N)-*Abaecin (C)*

CACCACCACCACCACCACTCTGACCAGGAGGCAAAACCTTCAACTGAGGACTTGGGGGATAAGAAGGAAGGTGAATATATTAAACTCAAAGTCATT
GGACAGGATAGCAGTGAGATTCACTTCAAAGTGAAAATGACAACACATCTCAAGAAACTCAAAGAATCATACTGTCAAAGACAGGGTGTTCCAATG
AATTCACTCAGGTTTCTCTTTGAGGGTCAGAGAATTGCTGATAATCATACTCCAAAAGAACTGGGAATGGAGGAAGAAGATGTGATTGAAGTTTATCA
GGAACAAACGGGGGGT***TATGTACCtcTACCAAAcGTtCCtCAACCAGGtCGTaGaCCtTTTCCtACcTTcCCTGGTCAGGGtCCtTTCAATCCaAAAAT***
cAAATGGCCTCAAGGCTAT

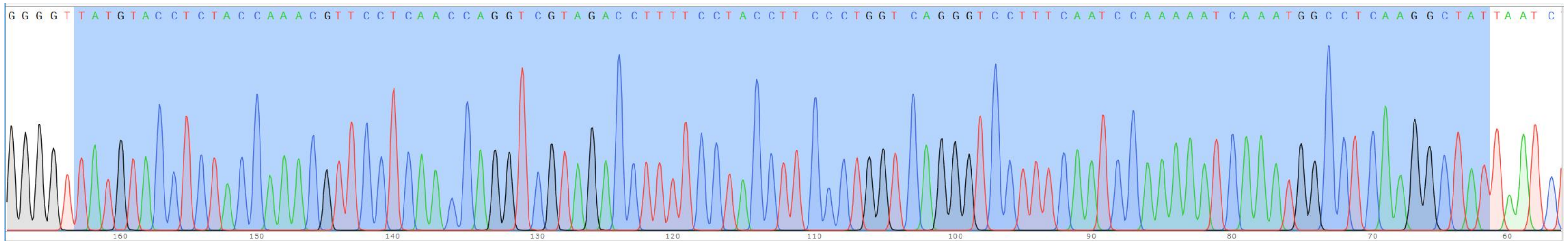

B Sequence for 6xHis(C) SUMO(C)-*Abaecin (N)*

CACCatCatCACCatCACTCTGatCAaGAaGCtAAACCTTCtACTGAaGatcttGGtGATAAaAAaGAAGGTGAATAcATTAAACTaAAAGTaATTGGtCAa
GATAGCAGTGAaATTCACTTCAAAGTaAAAATGAccACACATttgAAGAAAttaAAAGAAagtTACTGTCAAcgtCAaGGTGTTCCtATGAAcTCtttacgc
TTcttgTTTGAGGGTCAacgtATcGCTGATAATCAcACTCCAAAgGAACTtGGAATGGAGGAAGAAGAcGTaATTGAAGTTTATCAGGagCAAActGGc
GGT***TATGTACCATTACCAAATGTACCACAACCAGGACGTCGTCCGTTTCCAACGTTTCCTGGTCAGGGACCATTCAATCCGAAAATTAAAT***
GGCCTCAAGGCTAT

C Sequence for 6xHis(C) SUMO(C)-*Abaecin (C)*

CACCatCatCACCatCACTCTGatCAaGAaGCtAAACCTTCtACTGAaGatcttGGtGATAAaAAaGAAGGTGAATAcATTAAACTaAAAGTaATTGGtCAa
GATAGCAGTGAaATTCACTTCAAAGTaAAAATGAccACACATttgAAGAAAttaAAAGAAagtTACTGTCAAcgtCAaGGTGTTCCtATGAAcTCtttacgc
TTcttgTTTGAGGGTCAacgtATcGCTGATAATCAcACTCCAAAgGAACTtGGAATGGAGGAAGAAGAcGTaATTGAAGTTTATCAGGagCAAActGGc
GGT***TATGTACCtcTACCAAAcGTtCCtCAACCAGGtCGTaGaCCtTTTCCtACcTTcCCTGGTCAGGGtCCtTTCAATCCaAAAATcAAATGGCCTC***
AAGGCTAT

Fig. S1 Sequences of three different codon-optimized 6xHisSUMO-abaecin. 6xHis sequences were underlined, SUMO sequences are represented with standard font and the sequences of abaecin are italicized. N, native sequence; C, codon-optimized sequence. Sequence result for abaecin of 6xHis(N)SUMO(N)-Abaecin (C) construct, which was used for this study, is represented.
