# Supplementary material for: A new prokaryotic expression vector for the expression of antimicrobial peptide abaecin using SUMO fusion tag
Source: BMC Biotechnol. 2019 Feb 15;19:13. doi: 10.1186/s12896-019-0506-x (PMC6377777; doi:10.1186/s12896-019-0506-x)
Supplement: Supplementary file 2 — Table S1. Expression of antimicrobial peptides using E.coli and their subsequent release and purification. (PDF 73 kb) [file 12896_2019_506_MOESM2_ESM.pdf]

Supplementary table 1. Commonly used expression, peptide release and purification systems of antimicrobial peptides produced in *E.coli*

Expression			
Direct expression	Non-carrier bearing expression	• Large size and aggregation prone antimicrobial peptides are eligible<br>• His-tag is usually used for purification, and can block toxicity of antimicrobial peptides<br>o Refolding is time-consuming and may not guarantee recovery of activity	
Fusion expression	Solubility-enhancing carriers	GST (26.9 kDa)	• Rapid purification of the GST-fused protein from crude lysate<br>• Blocks toxicity to the host cell<br>o GST was highly susceptible to proteolytic degradation in several cases<br>o Reduced yield due to the large size of the GST due to low peptide-to-carrier ratio
		Thioredoxin (11.8 kDa)	• High yield due to high peptide-to-carrier ratio<br>• Chaperone-like activity can promote correct disulfide bond formation<br>• Blocks toxicity to the host cell and prevents proteolytic degradation of peptides<br>o Affinity tag such as His-tag is usually used for purification
		SUMO (11.2 kDa)	• High yield due to high peptide-to-carrier ratio<br>• Improves folding by the attachment of the carrier<br>• Peptide release by SUMO protease is both efficient and highly accurate, leaving no additional amino acids<br>• Blocks toxicity to the host cell and prevents proteolytic degradation of peptides<br>o Requires additional affinity tag such as His for purification
	Aggregation-promoting carriers	PurF fragment (16.3 kDa)/ PaP3.30 (17.6 kDa)/Ketosteroid isomerase (13.5 kDa)/ TAF12 domain (8.4 kDa)	• Rapid purification of fused peptides through insoluble expression<br>• Blocks toxicity to the host cell and prevents proteolytic degradation of peptides<br>o Requires refolding to restore activity
	Self-cleavable carriers	Intein (51.5 or 17.3 kDa) and N^{pro} systems	• Exogenous proteases or chemicals are not required to remove tag<br>• Facile purification of target proteins<br>o Reduced yield due to large size of intein/chitin domain<br>o Uncontrolled autocleavage of the intein fusions could cause toxicity to host<br>o Requires extensive dilution and long incubation time for N^{pro} system, and possible incomplete cleavage
	Secretion signals	Signal peptide	• Eligible expression of cysteine-rich antimicrobial peptides in periplasmic space where disulfide bond formation is favored<br>o Possible secretion of the peptide to culture media can be toxic to host

• advantages o disadvantages

Peptide release			
Chemical	CNBr	Met ↓	o Limited use of chemical reagents due to methionine presence within the target peptide<br>o Increased complexity and reduced yield of purification due to methionine presence within the target peptide<br>o Side chain modification by reaction buffers such as 70% formic acid
	Formic acid	Asp ↓ Pro	• Increased cleavage accuracy in comparison to CNBr due to residue pair recognition<br>o Esterification of serine and threonine residues by formic acid<br>o Modification of Asn or Gln to their hydroxamic acid forms by hydroxylamine
	Hydroxylamine	Asn ↓ Gly	
Enzyme	Enterokinase	AspAspAspAspLys ↓	o More expensive and more sensitive to pH and chaotropes in comparison to SUMO and TEV proteases
	Factor Xa	IleGluGlyArg ↓	
	Thrombin	LeuValProArg ↓ GlySer	
	SUMO protease	SUMO ↓	• Cleaved target peptide maintains native N-terminus, without unwanted amino acid attached because the SUMO protease recognize SUMO’s tertiary structure, rather than linear sequence<br>• Like the SUMO protease, TEV protease also can generate native N-terminus in many cases<br>• Urea-resistant property of the enzymes enables cleavage of peptides from the carriers in the presence of a small amount of urea which prevents protein aggregation and loosens compact structures<br>• Both enzymes can be manufactured in large quantities with relative ease compared to enterokinase, factor Xa and thrombin
	TEV protease	GluAsnLeuTyrPheGln ↓ (Gly/Ser)	
**Purification**			
• Released peptides are usually purified by high performance liquid chromatography (HPLC)<br>• Liberated peptides can also be more efficiently purified using cation-exchange chromatography due to the high PI values of antimicrobial peptides compared to carrier proteins			

• advantages o disadvantages

References

1. Li Y. Carrier proteins for fusion expression of antimicrobial peptides in Escherichia coli. Biotechnol Appl Biochem. 2009;54:1–9.
2. Li Y. Recombinant production of antimicrobial peptides in Escherichia coli: a review. Protein Expr Purif. 2011;80:260–7.
3. Malakhov MP, Mattern MR, Malakhova OA, Drinker M, Weeks SD, Butt TR. SUMO fusions and SUMO-specific protease for efficient expression and purification of proteins. J Struct Funct Genomics. 2004;5:75–86.
